# 3D-Printed Components for Cost-Effective Polarisation Sensing of Terahertz Radiation †

**DOI:** 10.3390/s25237106

**Published:** 2025-11-21

**Authors:** Adrianna Nieradka, Mateusz Kaluza, Paweł Komorowski, Agnieszka Siemion

**Affiliations:** 1 Faculty of Physics, Warsaw University of Technology, Koszykowa 75, 00-662 Warsaw, Poland; mateusz.kaluza@pw.edu.pl (M.K.); agnieszka.siemion@pw.edu.pl (A.S.); 2 Institute of Optoelectronics, Military University of Technology, gen. Sylwestra Kaliskiego 2, 00-908 Warsaw, Poland; pawel.komorowski@wat.edu.pl

**Keywords:** terahertz radiation, polarisers, optical components, 3D printing, polymer composites, fill factor, degree of polarisation

## Abstract

This study covers the research on 3D-printed structures for the polarisation sensing in the terahertz (THz) frequency range. Proposed polarisers can be combined with various detectors to obtain cost-effective and easily accessible polarisation-sensitive sensors. Multiple commercially available filaments for 3D printing with various additives were used to obtain good-quality, efficient optical components. Fused deposition modelling (FDM) was selected as the manufacturing technique due to the reliable and repeatable results of 3D printing technology. The research focused on elements with sub-THz features designed to determine the polarisation of incident radiation. Manufactured polarisers have been tested in two setups, verifying narrow-band operation at the design wavelength as well as broad-band operation across the THz spectrum. Both setups allowed the change of the angular position of the examined elements around the optical axis. The final results proved the possibility of obtaining cost-efficient polarisers functioning comparable to the commercially available wire-grid polarisers. Constructive conclusions were drawn to determine the proper materials, their additives, the chosen fill factors (FFs), and the dimensions of the polarisers, ensuring optimal performance and efficiency in manipulating THz radiation.

## 1. Introduction

Techniques employing terahertz (THz) radiation have undergone remarkable growth in recent years. The unique properties of this radiation range allow for a wide range of applications in telecommunications [1,2,3,4,5], security systems [6,7,8,9], or setups for beam-shaping [10,11,12,13,14,15], which is inseparable from beam polarisation analysis [16,17,18,19,20].

The polarisation of the radiation is of fundamental importance in both classical and quantum optics. Today, polarisers are widely applied in liquid crystal displays (LCDs) [21,22,23], photography [24], glasses for three-dimensional (3D) displays [25], or sunglasses [26], demonstrating their importance in many aspects of life.

The operation of a THz wire-grid polariser relies on introducing strong anisotropy in the interaction between the incident electromagnetic wave and a subwavelength periodic structure. This effect is achieved through a regular array of parallel conductive or metal-enhanced wires that selectively transmit the electric-field component perpendicular to the wire orientation, while suppressing the parallel component through induced surface currents. The key geometrical parameters, such as period, wire width, fill factor, and wire height, must remain well below the design wavelength to ensure proper polariser operation and high extinction performance [13,20,27]. The relatively long THz wavelengths permit subwavelength structures to be realised in larger scales, making fabrication feasible using accessible methods, like 3D printing. The fast accelerating development of the THz techniques justifies the prediction of the need for cost-effective and easy-to-manufacture polarisers for the THz waves.

This scale advantage has motivated investigations of wire-grid structures across different wavelength ranges [27], where the influence of substrate material for wavelengths between 20 µm and several hundred micrometres was analysed, and another [28], where these observations were extended further into the THz spectrum. These works collectively reinforce that subwavelength periodicity and conductive-wire geometry remain the fundamental design principles governing the operation of THz wire-grid polarisers. Beyond classical wire-grid layouts, alternative THz polarisation-control devices have been demonstrated in recent years. Examples include metasurface-based [29,30,31] converters, which enable advanced manipulation of the polarisation state, including multifunctional linear-to-cross and linear-to-circular transformations [32] as well as achromatic waveplates exploiting engineered form-birefringence [33] and stacked birefringent dielectric-plate waveplates capable of broad bandwidth polarisation conversion in the THz regime [34]. Although such components can achieve outstanding performance, their fabrication typically requires micro- and nanolithography, clean-room processing, or advanced material engineering [35], which may limit accessibility for broader THz applications. On the other hand, the development of 3D printing techniques opened up a wide spectrum of possibilities to produce highly efficient and low-cost THz optical components using fused deposition modelling (FDM) technology [36,37,38,39] and commercially available filaments. The relatively long wavelength of the radiation in the THz band makes the resolution of even very accessible FDM 3D printers sufficient to meet the requirements of manufacturing efficient THz optical components.

In this context, additive manufacturing provides an attractive and highly accessible alternative for the sub-THz range, where the required feature sizes are compatible with standard FDM printers. By adjusting structural parameters and material composition, printed wire-grid structures can achieve polarisation filtering without specialised fabrication facilities.

This study aims to investigate the feasibility of manufacturing affordable and accessible THz polarisers using the FDM 3D printing method. The goal is to provide low-cost devices that can be readily tailored to diverse THz detectors to ensure robust polarisation sensitivity. A general outline of the research activities and preliminary results was presented at the MIKON2024 Conference [40]. This paper vastly extends that publication, providing crucial details on the design of the polarisers, the range of applicable materials, the recommendations for 3D printing parameters, and lastly the experimental results obtained using various polarisers and frequency bands. The performance of the manufactured polarisers depends not only on the filament used but also on the size of the component’s details. The variety of samples allowed for a focused analysis of the fill factor (FF) and experimental repeatability. Analysis of the performance of polarisers was carried out in different experimental scenarios, providing detailed angular and spectral dependencies.

## 2. Methodology

The polarisation state of the system can be affected using an external optical element such as a linear polariser [41]. The wire-grid polariser (WGP) used as a reference in this research has a periodic structure consisting of thin conductive wires. Such a grid, consisting of alternating dielectric (surrounding—air) and conductive (wires—metal) areas, must have a period matched to the design wavelength at which it is intended to operate. Electromagnetic (EM) waves polarised perpendicular to the wires interact with grid lines whose wire width is insufficient to excite electron motion. It results in a partial reflection of the energy, and the remaining energy passes through the surface. Such constructed polarisers behave like a dielectric. On the other hand, the propagating EM waves polarised parallel to the wires cause the movement of electrons in that direction. Thus, the EM wave is reflected from the surface, and the polariser in this case behaves like a metallic surface, also causing a minimal loss of energy due to the heating of the wires [27,42].

In this study, the parallel ($t_{\|}$) and perpendicular ($t_{⊥}$) polarisation transmittances were obtained from experimental measurements. The data were collected using two measurement systems: one employing a monochromatic source and the other operating over a broad-band THz spectral range. The use of two complementary setups enabled a substantive comparison of the obtained results.

### 2.1. Materials

Manufacturing components using 3D printing has become increasingly popular in recent years in various areas of research, starting from mechanical components [43] and extending to body prostheses [44]. At the same time, advances in 3D printing technologies are evident in the development of efficient and affordable passive optical components in the THz range [45].

To correctly design a THz passive optical component, it is essential to select a 3D printing filament with appropriate optical properties [46,47]. Therefore, the examination of optical properties of selected filaments was performed using a THz time-domain spectroscopy system (THz–TDS)—the TeraView TeraPulse Lx Modular System (TeraView Ltd., Cambridge, United Kingdom). Therefore, a set of samples was manufactured from the chosen polymeric materials and polymer composites using 3D printing with FDM technology. All the samples and polarisers presented in this study were manufactured using a Prusa MK3S+ 3D printer (Prusa Research a.s., Prague, Czech Republic), which guarantees a horizontal positioning accuracy of 10 µm and a vertical accuracy of 2 µm. Polymers analysed include Cyclic Olefin Copolymer (COC) from the Swiss company Creamelt, as well as Acrylonitrile Styrene Acrylate (ASA) provided by Rosa3D. Two polymer composites with a Polylactic Acid (PLA) base were also studied: the first, PLA with copper powder, commercially named MetalFil Classic Copper by FormFutura, and the second, PLA with a powdered steel additive, commercially known as SteelFil by ColorFabb. Additionally, two PETG-based composites were examined: PETG MDT (PETG with magnetically detectable metal fillers by Smart Materials 3D) and CarbonFil (PETG reinforced with carbon fibres by FormFutura). To broaden the comparison, the XT-CF20, a co-polyester reinforced with approximately 20% carbon fibre by ColorFabb, was also evaluated. For clarity, the polymer base names will be used throughout this study.

**Figure 1:**
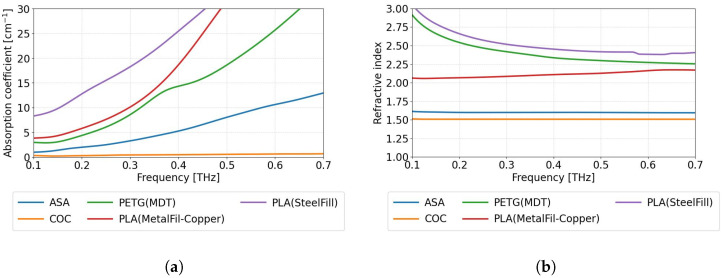
The dependence of optical properties on the THz frequency for selected 3D-printable materials. (**a**) The absorption coefficient and (**b**) the refractive index.

The optimal dimensions of the cylindrical samples for the THz-TDS experiments were a thickness of 4 mm and a diameter of 13 mm. Figure 1a presents the absorption coefficient, and Figure 1b presents the refractive index values of the samples in the frequency domain ranging from 0.1 THz to 0.7 THz. The results for PETG (CarbonFil) and XT-CF20 are not shown, as these materials exhibited too high absorption to detect the signal using the transmissive THz-TDS measurement method.

In most cases, the absorption coefficient (Figure 1a) increases significantly with frequency. It is worth noting that COC has a relatively low and almost constant absorption coefficient, with a refractive index approximately equal to 1.51 in the considered range (Figure 1b), making it one of the most suitable materials for prototyping transparent THz optical components. A material with almost the same low refractive index as COC is ASA; however, it exhibits a significantly higher absorption coefficient. Both PLA composites (MetalFil-Copper and SteelFill), as well as PETG (MDT), possess high absorption coefficient values even for the low THz frequencies with a steep increase with the increasing frequency. The refractive index values for these materials are also relatively high, with a noticeable dispersion characteristic. The diversity of optical properties of the described filaments enables their use in a wide range of applications [36,39].

### 2.2. Polarisers

**Figure 2:**
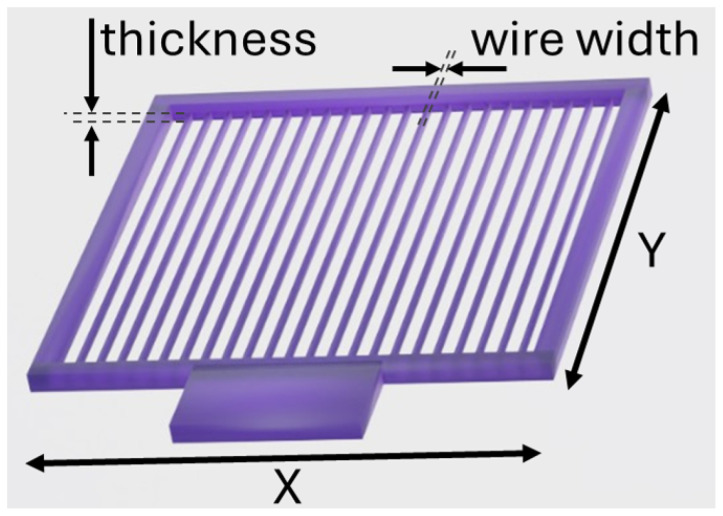
Model of the P22 THz polariser with indicated geometric parameters, individually adjusted to the design requirements. The parameters, such as wire width, thickness, and overall dimensions, are discussed in the text and summarised in Table 1.

The presented research focused on twenty-six customised polarisers. Figure 2 illustrates a model of polariser called P22, in which the geometric parameters are marked and tailored to the specific design requirements. Their parameters such as material specification and dimensions of the individual component, the wire width, and its thickness, as well as the theoretical and real fill factors (FFs) of the polarisers, are outlined in Table 1. The manufactured elements, due to their specifications, were divided into four series. All components were designed for a relatively low frequency of 95.7 GHz, ensuring a subwavelength size of details.

**Figure 3:**
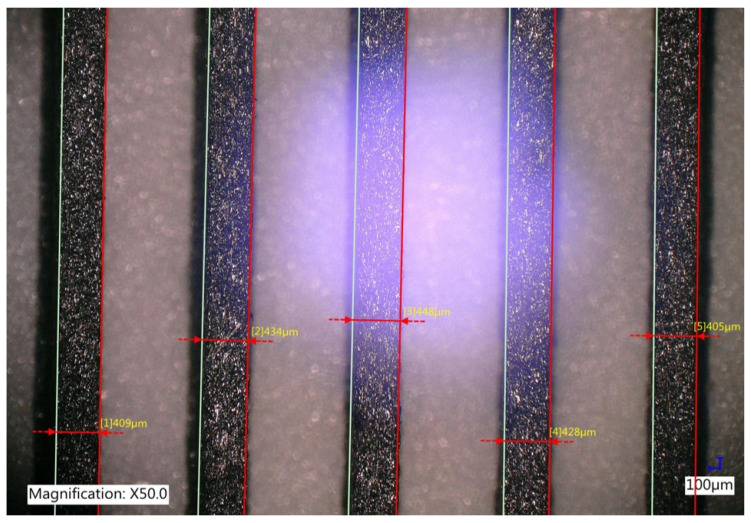
Wire width measurements performed on the polariser manufactured from XT-CF20 material using FDM technology.

The designed polarisers were printed using the FDM 3D printer with a nozzle diameter of 400 μm. Due to the properties of the materials, such as the solidification time, the shrinkage of the material, and the gradation of the additives, the actual rectangular shape of the wires was verified with a microscope, as shown in Figure 3. The thickness of the polarisers for the first, second, and third series was approximately 1 mm. The thickness of the fourth series varied. Despite the identical thickness across the three series, the mismatch between theoretical and actual wire widths differed as a result of the materials used, which in turn affected the measured real FF.

**Figure 4:**
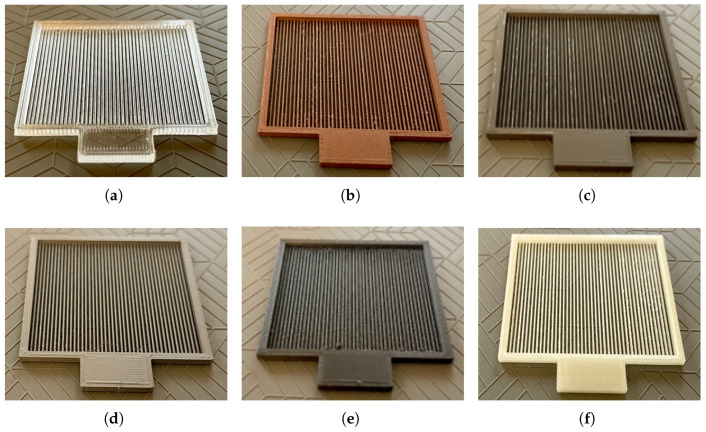
3D-printed polarisers with dimensions of 5 cm × 5 cm. (**a**) P1—COC (material highly transparent in the THz band). (**b**) P3—PLA (MetalFil–Copper), a composite of PLA with copper powder. (**c**) P4—PLA (SteelFil), a composite of PLA with a steel additive. (**d**) P5—PETG (MDT), PETG with ferromagnetic additives. (**e**) P6—PETG (CarbonFil), PETG with a carbon additive. (**f**) P8—ASA (uncoated); the same material also served as the base for P2 (ASA sprayed with Mirror Mist) and P7 (ASA sprayed with Forever Paints) prior to coating.

The first series of polarisers varied in the type of material used for their manufacturing. Figure 4 demonstrates six polarisers with square surfaces (5 cm × 5 cm) from the first printed series. The polariser in Figure 4a (P1) was printed from COC, a material that is highly transparent in the THz band. Due to its geometry, consisting of alternating regions of COC filament and air, the structure may exhibit a form birefringence characteristic of thin grating-based waveplate-like elements in the THz range [33]. Nevertheless, in the context of this study, it is referred to as a polariser for consistency within the printed series. Figure 4b–e present polarisers labelled from P3 to P6 manufactured with PLA (MetalFil–Copper), PLA (SteelFil), PETG (MDT), and PETG (CarbonFil), respectively. Figure 4f shows the polariser P8 manufactured from ASA (uncoated). The same material also served as the base for P2 (ASA sprayed with Mirror Mist) and P7 (ASA sprayed with Forever Paints) prior to coating. The sprays had a high percentage content of pure zinc and aluminium to increase the reflection of the incident beam [14,15]. In these components, the metallic coating was applied only to the side intended to be illuminated in the experimental setup, providing a single-surface reflective treatment without altering the remaining parts of the structure.

The second series of polarisers was printed with varying FFs to check the impact of this parameter on the performance of the polarisers. This series and the remaining two were printed using the single material XT-CF20. However, the third and fourth series used smaller 3 cm × 3 cm formats. This downscaling reduced the deformation of the wire and the defects in the polarisers.

The third series of polarisers was printed with the same range of FFs as in second series. Although, since the second series delivered polarisers with real FF values lower than the planned theoretical ones, the third series of polarisers was supplemented with polarisers with real FFs higher than 53%, specifically of 60% and 70%. The smaller size of the polarisers increased mechanical stiffness and enabled measurements over a broader THz frequency range, extending beyond the design wavelength.

The fourth and last series of polarisers P23–P26 with a real FF of 38% were used to investigate the performance comparison in a broad spectral range between the same wire width of 440 μm but different wire thicknesses of 0.5 mm, 1 mm, 1.5 mm, and 2 mm.

## 3. Experimental Evaluation

**Figure 5:**
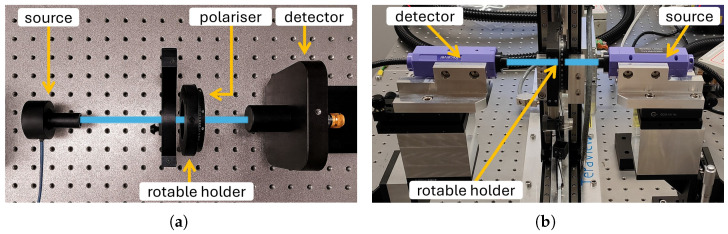
The experimental setups used to measure the signal transmission dependent on the angular position of the polarisers placed perpendicularly to the radiation beam. (**a**) The first setup for the narrow-band verification used an IMPATT diode-based source. (**b**) The second setup for the wide-band verification used the THz-TDS system.

Two experimental setups were assembled to thoroughly investigate the angular and spectral aspects of the polarisers’ performance. The photographs of the setups with the description of their elements are presented in Figure 5.

Figure 5a presents the first experimental setup that was used to verify the dependence of the transmittance of the polarisers on its angular position ranging from 0° to 180° for the chosen frequency of 95.7 GHz. This setup consisted of the TeraSense impact ionisation avalanche transit-time (IMPATT) diode as a radiation source, a polariser-under-test located on the rotary holder with marked angles, an optical chopper, and a pyroelectric detector (THZ10 by SLT) connected to a Tektronix MSO44 oscilloscope.

The second setup, presented in Figure 5b, was used to analyse the performance of printed polarisers in a broader frequency spectrum, using the TeraView TeraPulse Lx THz-TDS system. It should be noted that the original parameters of the designed polarisers were optimised for the frequency of 95.7 GHz. Nevertheless, it can be expected that their operating frequency band is wider, which was intended to be verified in this part of the experiment.

In both experimental setups, the emitted radiation is highly linearly polarised.

The description of the experimental results has been divided into two subsections according to the two experimental setups described above.

### 3.1. Narrow-Band Operation

**Figure 6:**
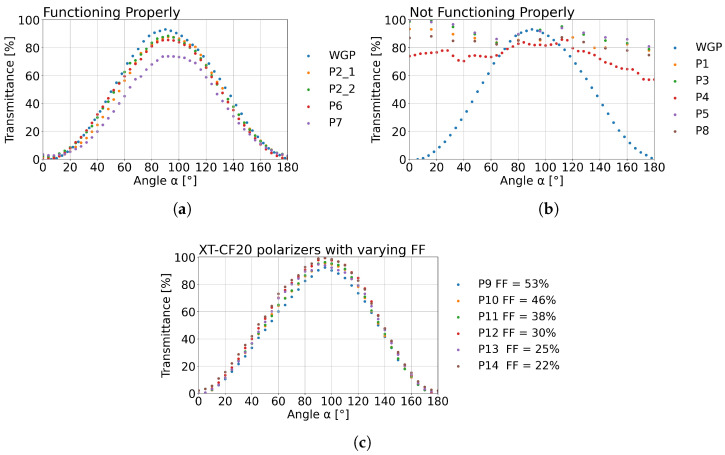
The normalised transmittance calculated from the signal detected behind the polarisers. (**a**) The results of measurements of the first series of polarisers made of different materials (where reference is the commercial wire-grid (WGP) polariser) with an example of the manufacturing repeatability with the use of measurements for the polariser P2 shown as $P2_{1}$ and $P2_{2}$. (**b**) The results of measurements of the first series of polarisers made of different materials that are not operating correctly. (**c**) Results of measurements of the second series of polarisers P9–P14 manufactured from XT-CF20 and designed with different FF values.

During the first experiment, the focus was on the first and second series of polarisers. Polarisers’ transmittance measurements were taken every 5° between the angular position of 0° and 180°. The rotation occurred perpendicular to the optical axis defined by the propagation direction of the THz beam. The detected signals were gathered into detailed diagrams that were normalised and plotted in Figure 6.

In Figure 6a, the reference is a commercially available WGP from MicroTech Instruments. Its performance is compared with the following: three polarisers sprayed with mirror paint $P2_{1}$, $P2_{2}$ (Mirror Mist) and P7 (Forever Paints), as well as with the polariser P6 made of PETG with 20% of carbon fibre additive. $P2_{1}$ and $P2_{2}$ are presented separately to investigate the repeatability of the manufacturing process of the 3D-printed polariser. For an angle of around 90°, the maximum transmittance value obtained for the reference polariser was 93.2%. For polarisers $P2_{1}$, $P2_{2}$, P6, and P7 it was equal to 87.8%, 88.5%, 85.6%, and 73.9%, respectively. It should be highlighted that the transmittances obtained for duplicated polarisers $P2_{1}$ and $P2_{2}$ differ only by 0.7 percentage points, which corresponds to a less than 1% difference in the measured values. This shows that even without specifically defined manufacturing and handling procedures, the repeatability of the fabrication is relatively high. Most of the results in Figure 6a are consistent and meet the design targets, but the P7 polariser stands out significantly. Its transmittance differs by 19.3 percentage points relative to the reference WGP. P7 uses the same ASA base as $P2_{1}$ and $P2_{2}$; the discrepancy is attributable to the Forever Paints spray coating, which led to the observed performance deviation.

Figure 6b presents the results obtained for the polarisers that performed significantly worse than the reference WGP. Polarisers P1, P3, P5, and P8 show similar wave-like behaviour with high transmittance across the entire angular range investigated. This result was expected for polarisers P1 and P8 as the materials of these components are characterised by a low absorption coefficient and a low refractive index. On the other hand, the P3 and P5 polarisers seemed very promising in the design stage, as they were manufactured from the filaments with metal additives. However, the experimental results indicate that they are unsuitable for fabricating polarisers. Even less predictable behaviour was observed for the P4 polariser made from PLA(SteelFill), which might be due to the low amount of metal in the composite. The natural conclusion is that these materials do not form long enough conductivity chains in the 3D-printed wires to allow for the induction of electrical currents.

Figure 6c presents the results obtained for the second series of polarisers, for which the specifications are given in Table 1. In Figure 6c polarisers differ by the FF values. From the transmittance values obtained for the 90° angle, it can be determined that the transmittance increases as the FF decreases. An exception is the P13 polariser with an FF equal to 25%. Thus, the best transmittance result of 99.6% was obtained for the P14 polariser with an FF equal to 22%. The lowest transmittance result of 95.9% was obtained for the P9 polariser with an FF equal to 53%. Nevertheless, all of the polarisers investigated in this part are very promising, losing less than 4.1% of an input signal.

Furthermore, based on the transmitted values obtained, the degree of polarisation (DOP), determined using Equation (1), was calculated to assess the efficiency of the polarisers (P9–P14).

(1) $$DOP=\frac{t_{⊥}-t_{\|}}{t_{⊥}+t_{\|}}.$$

The DOP is a key parameter for describing optical systems that are sensitive to the state of polarisation of radiation. This parameter ranges from 0% when the radiation is not polarised to 100% when the radiation is fully polarised, which means that all waves oscillate in a single plane [48]. The negative value of DOP indicates polarisation in the opposite direction than expected. The DOP values obtained from the second series of polarisers for the 90° angle are presented in Table 2.

The highest DOP values, above 86%, were obtained for polarisers P10–P13. All calculated DOP values exceeded 83%, which is comparable to or higher than the typical performance of commercially available metal wire-grid polarisers. This confirms the reliable operation of the proposed optical components.

### 3.2. Broad-Band Operation

**Figure 7:**
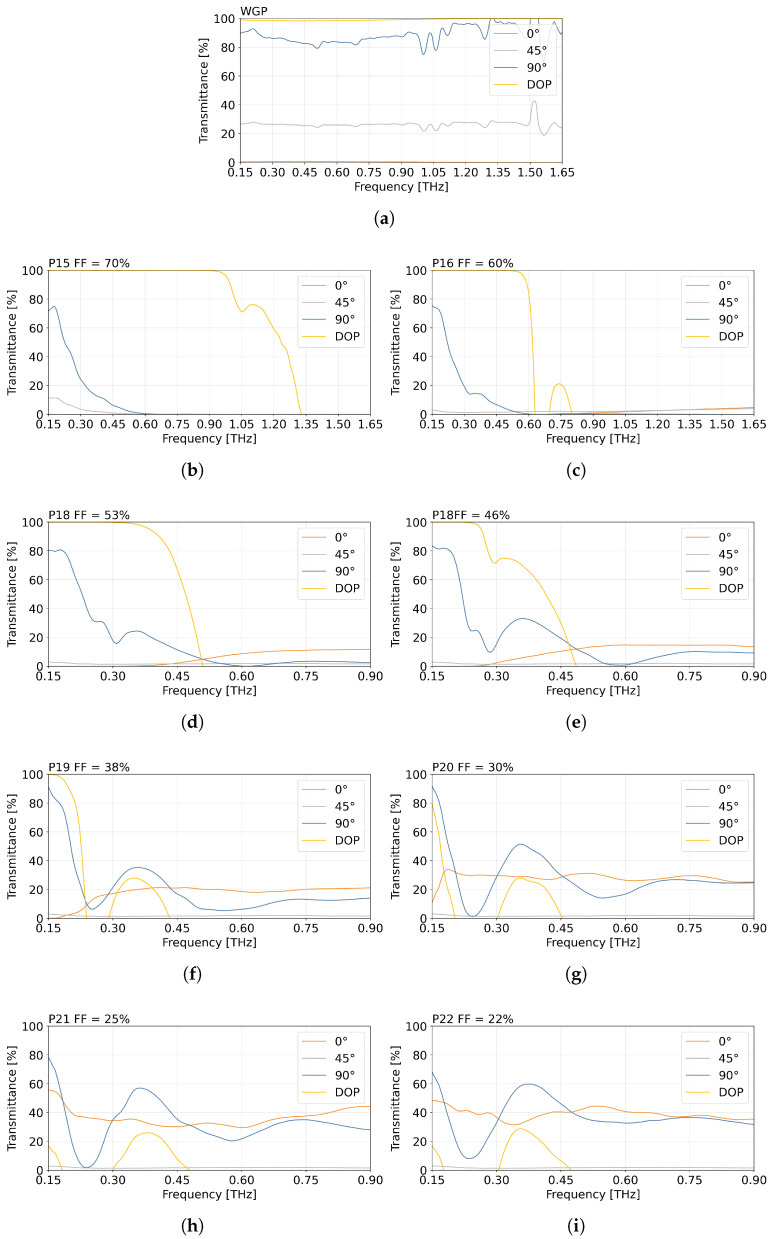
Normalised dependence of the degree of polarisation (DOP) and the transmittance at 0°, 45°, and 90° angles as a function of THz radiation frequency for (**a**) a commercial wire-grid polariser, and polarisers P15–P22, manufactured from XT-CF20 with the following FF values: (**b**) 70%, (**c**) 60%, (**d**) 53%, (**e**) 46%, (**f**) 38%, (**g**) 30%, (**h**) 25%, and (**i**) 22%.

Broad-band frequency-dependent transmission measurements were carried out using the THz-TDS system. Measurements were performed for the third and fourth series of polarisers manufactured from composite XT-CF20. XT-CF20 was selected for the manufacturing of polarisers for this part of this study due to the addition of carbon fibre. This type of composite demonstrated the best performance in the previous section of the experimental measurements. This material also allowed the printing of the 3D model of the polarisers to be more precise compared to the PETG composite. As a result, due to the lower gradation of the carbon fibre in the composite, there was less roughness in the printed objects and a smoother finish to the polariser wires. The third series of optical objects is characterised by different real FF values equal to 22%, 25%, 38%, 46%, 53%, 60%, and 70%. The results obtained for polarisers marked as P15–P22 as well as measurements of the commercial WGP to provide a reference for comparison are presented in Figure 7. Moreover, the normalised DOP and transmittance for selected frequencies are presented in Table 3 and Table 4.

Figure 7 presents the transmittance of the reference polariser WGP and manufactured polarisers with different FF values. As expected for a highly precision-manufactured element, the WGP shows higher transmittance and DOP stability at higher THz frequencies. Figure 7i shows that the high DOP value is maintained over an increasingly wide frequency range as the FF value increases. For P19 (FF = 38%), this value is greater than 70% for values as high as 0.22 THz, extending the range over 124 GHz further than the frequency for which the polarisers were designed (95.7 GHz). For P16 and P19 the result of 100% DOP is achieved up to 0.53 THz and 0.92 THz, while allowing for 70% DOP till 0.62 THz and 1.16 THz, respectively. Polarisers P21 and P22 resulted in a significantly lower DOP compared to polarisers P15–P20. Across the analysed frequency range, their DOP values did not exceed 32% and 21%, respectively.

The performance of polarisers P17 and P18 with an FF of 46% and 53% had already been discussed in a conference paper [40]. Overall comparison between all FFs allows one to conclude that the larger the FF, the greater the frequency range for which the DOP is high. This dependence can be seen in more detail in Table 3.

Table 3 presents the normalised DOP values of the analysed polarisers with selected FFs. The results obtained have been separated by colour to allow for easier identification of the results. The green colour represents a DOP greater than 98%, yellow is assigned to DOP values of at least 70%, orange indicates positive values above 0% but below 70%, and red denotes negative DOP values. These results for selected frequencies demonstrate the highly effective performance of the polarisers for broad-band operation.

Table 4 summarises the perpendicular transmittance $t_{⊥}$ [%] of the polarisers analysed for selected FFs and frequencies. Values are colour-coded to aid in interpretation: green represents transmittance equal to and above 80%, yellow is transmittance from 70% to 79%, orange represents transmittance from 20% to 69%, and red represents when transmittance is below 20%.

Comparison of Table 3 and Table 4 allows a more precise evaluation of the performance of the polarisers. The most promising values are observed for polarisers P17–P20 at 150 GHz. For P15 and P16, the results clearly demonstrate that, despite DOP approaching 100%, the absolute transmittance remains low. This emphasises how important it is to pay attention to both of these parameters. Compared with the WGP, the performance of the printed polarisers varies more strongly with frequency, which is consistent with their fabrication method and the sensitivity of their geometry to FF.

In Figure 7 for the position of 90°, it can be observed that P15, P16, P17, and P18 show similar trends in the decrease in the transmittance in the range of 0.15–0.23 THz. Polarisers P19, P20, P21, and P22 also show an analogous transmittance shape behaviour. In the case of polarisers with lower FF, after a decrease, a peak of increase in transmittance is observed around the frequency of 0.35 THz. However, subsequently, it decreases again as the frequency of radiation increases. In contrast, the WGP demonstrates a smoother and more stable transmittance response over the same range, which further highlights its role as a reference for evaluating the frequency-dependent behaviour of the printed polarisers.

**Figure 8:**
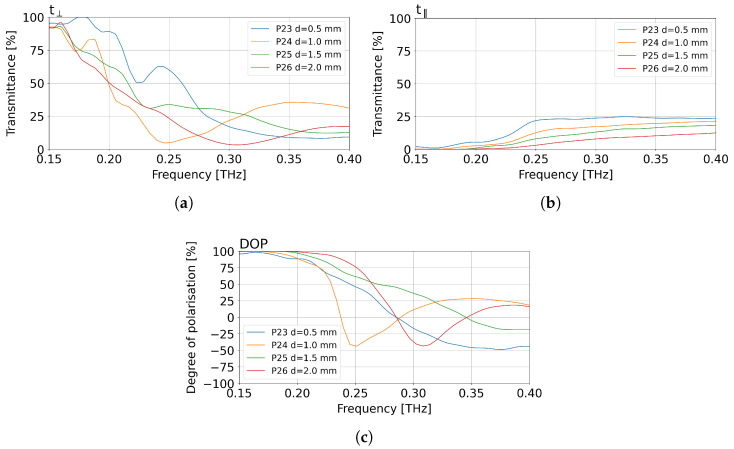
Normalised measurements results for polarisers P23–P26, made from XT-CF20 with a carbon fibre additive, with FF = 38% and different thicknesses *d* of 0.5 mm, 1 mm, 1.5 mm, and 2 mm. (**a**) Normalised transmittance for the transmissive angular position of the measured polarisers. (**b**) Normalised transmittance for the blocking angular position of the measured polarisers. (**c**) Normalised degree of polarisation (DOP) for the measured polarisers.

Subsequently, for the performance of the fourth series of polarisers with thicknesses equal to 0.5 mm, 1 mm, 1.5 mm, 2 mm, and FF = 38%, each was investigated in the THz-TDS system. The measurements covered the range from 0.15 THz to 0.40 THz, as shown in Figure 8.

Figure 8a presents the normalised transmittance for the transmissive angular position of the polarisers. Between the components examined, a similar degree of decrease in value can be observed as the frequency increases. The most rapid decrease in value can be found for the P24 polariser with a thickness (*d*) of 1 mm, in the frequency range from 0.15 THz to 0.25 THz. A similar decrease in the frequency range from 0.15 THz to 0.23 THz is characteristic for the polarisers P25 and P26 (*d* equal to 1.5 mm and 2.0 mm, respectively). The P26 polariser manifests the smoothest characteristics. Its lowest transmittance value was recorded after the frequency of 0.3 THz. The largest wavelike behaviour of the signal is observed for the P23 polariser, which is the result of unwanted reflections occurring in the THz-TDS system due to the thin thickness of the optical element.

Figure 8b demonstrates normalised transmittance for the blocking angular position of the measured polarisers. The characteristics confirm the observations on the wavelike behaviour of the P23 polariser. The measurement results for the polarisers P24 and P25 again show similarities in transmittance values within the entire 0.15–0.4 THz range. The polariser P26 presents the highest efficiency, that is, the lowest transmittance over the whole measured range for the blocking angular position. This result can be attributed to the greatest thickness of the polariser. In addition, in the range 0.15–0.2 THz, all three of the four polarisers with higher thickness (P24–P26) manifest close to zero transmitted values.

The normalised dependence of DOP in Figure 8c indicates the efficiency of the performance of a given optical element. After analysis of the plots in Figure 8a,b, as expected the least promising is the thinnest polariser P23. The most promising result is obtained for the thickest polariser P26. Nevertheless, all four investigated polarisers show correct performance in terms of DOP (approaching 100%) for low frequencies.

## 4. Discussion

This paper focused on 26 custom polarisers, demonstrating that additive manufacturing can produce high-performance wire-grid polarisers for the sub-THz region, where the strongest performance is obtained below 0.2 THz. Each polariser designed for a low frequency of below 0.1 THz was designed according to its given parameters, assuring the subwavelength size of components. Subsequently, the polarisers were manufactured using a cost-efficient FDM method and examined in two experimental optical setups of single and a wide range of frequencies, providing promising results comparable to those of commercial WGP.

The first series of polarisers was manufactured using different types of polymeric materials and composites. Some of the polarisers were additionally covered with metallic coating, increasing their degree of reflection. The results obtained demonstrate that the use of a mirror spray and composites containing carbon fibre additives in the manufacturing process, which exhibit strong absorption in the THz radiation range, leads to the high performance of the produced optical elements. These doped or sprayed polarisers significantly outperform the other polarisers investigated in this series. The measured value of the transmittance in the angular position range of 0° to 180° of the tested polarisers was comparable to the commercially available WPG. In addition, the repeatability of the manufactured polarisers was also investigated, resulting in an insignificant difference between the measured transmittances of only 0.7%.

The second series of polarisers was printed from XT-CF20 material with approximately 20% carbon fibre. The dependence of the FF on the angular position of the component was investigated. The transmittance value of the manufactured polarisers varied only by up to 4.1% from the transmittance value obtained with commercial WGP. Moreover, all the calculated DOP values exceed a minimum of 83%, confirming that the polarisers are operating properly.

The third and fourth series were adjusted in the dimension size for better performance. Measurements in this series were conducted over a broader range of low THz frequencies using the THz-TDS system. The results indicate that properly designed polarisers with a specified high FF of 70% exhibit DOP as high as 100% in the broad THz frequency range from 0.15 THz to above 0.9 THz. However, the obtained data confirmed the assumption that DOP is not the only significant parameter when it comes to polarisers. This study also included in-depth information on the amount of signal transmitted perpendicularly to the orientation of the wires ($t_{⊥}$). This part of the experiment showed that the best performance for the frequency that exceeded the design frequency was obtained with polarisers with an FF equal to 30%, 38%, 46%, and 53%.

The fourth series showed that not only the material type used in the manufacturing process and the FF but also the thickness of the designed component are crucial factors in the design of the polariser. It should be emphasised that even if a highly absorptive material is used, such as XT-CF20, the thickness of the polarisers still has a significant impact on polariser performance. The research demonstrated that excessively thin polariser wires may degrade performance when the wires cannot fully reflect an incident beam. Other unwanted behaviour, such as internal reflections, may also appear inside the wires, causing an unreliable way of operating the optical component.

Approach to improving printed device performance is the optimisation of structural dimensions. The fourth series illustrates this, showing that modifying wire thickness can effectively tune the optical response. Optimisation of structural dimensions can lead to improved performance of printed devices. The fourth series illustrates this, showing that modifying wire thickness can effectively tune the optical response. Considering these findings, it is also relevant to examine potential improvements related to printing precision. Increasing the printing resolution by using a nozzle with a smaller diameter could enable the fabrication of wires that more closely match the theoretical design, which may yield FF values that better reflect the intended geometry. Achieving such precision would require careful optimisation of printing parameters, including extrusion temperature and print speed. Although a smaller nozzle diameter, such as 100 μm, may improve device performance, it would also considerably extend fabrication time. In this study, a 400 μm nozzle was selected due to it being widely accessible, and it ensures stable printing behaviour and offers a practical balance between print quality and production efficiency. Beyond improvements in printing precision, further enhancement may also be achieved through optimisation of the wire geometry itself.

In the broader context of THz polarisation control, many approaches have been explored in recent years, including metasurface-based devices and engineered birefringent structures. Although these concepts differ substantially in geometry and fabrication requirements, they share the common objective of shaping the polarisation state of THz radiation. The present results therefore provide a complementary perspective by demonstrating how additive manufacturing can implement this general principle in a fully accessible, sub-THz compatible form, which may support or motivate future investigations involving alternative geometries or design methodologies.

## 5. Conclusions

Comparing all scans performed using the two measurement setups, the best performing polariser would have an FF of approximately 50%, wire height of at least 1 mm, and could be made of materials such as ASA sprayed with Mirror Mist, PETG (CarbonFil), or XF-CF20. The best performance has been observed for the polarisers named in this article, namely P2, P6, and P17.

The presented research proves that it is possible to obtain good-quality and cost-effective 3D-printed polarisers. The achievement of very efficient optical elements is possible using commercially available filaments with various additives. This method gives the opportunity for further research on THz polarisers and provides easy access to THz optical components and enables further exploration of tailored design.

For broader context, the commercially manufactured WGP included in this study serves as a stable reference. Its transmittance and DOP characteristics remain highly uniform across the analysed THz range, which reflects the precision of its fabrication. The 3D-printed polarisers, by contrast, exhibit frequency-dependent behaviour closely linked to their fill factor and geometric parameters, offering tunability that can be tailored through design choices. Therefore, the comparison with the WGP highlights the complementary nature of both approaches: the stability of a precision-manufactured component and the flexibility and customisability enabled by additive manufacturing.

Using commercially available filaments with functional additives enables highly efficient optical elements. This approach supports 3D-printed structures for polarisation sensing in the THz range, as the proposed polarisers can be paired with a wide variety of detectors to form affordable, easily deployable, polarisation sensitive sensors. The goal was to deliver low-cost devices that can be readily tailored to different THz detectors while maintaining robust polarisation sensitivity, thereby opening clear avenues for future development and application.

## Figures and Tables

**Table 1:** Material composition, width of wires, and fill factor parameters of the printed polarisers.

No. of Polariser	Base Material	Admixture/ Spray Composite	Dimensions XY [cm]	Wire Width/ Thickness [mm]	Theoretical FF [%]	Real FF [%]
P1	COC	–	5 × 5	0.50/1.0	40	48
P2	ASA	Sprayed with Mirror Mist		0.44/1.0	40	42
P3	PLA (MetalFil-Copper)	Copper additive		0.47/1.0	40	42
P4	PLA (SteelFil)	Steel additive		0.45/1.0	40	45
P5	PETG (MDT)	Ferromagnetic additive		0.50/1.0	40	43
P6	PETG (CarbonFil)	Carbon additive		0.57/1.0	40	48
P7	ASA	Sprayed with Forever Paints		0.44/1.0	40	54
P8	ASA	–		0.44/1.0	40	42
P9	XT-CF20	Carbonadditive	5 × 5	0.44/1.0	70	53
P10					60	46
P11					50	38
P12					40	30
P13					33	25
P14					29	22
P15	XT-CF20	Carbonadditive	3 × 3	0.44/1.0	92	70
P16					79	60
P17					70	53
P18					60	46
P19					50	38
P10					40	30
P21					33	25
P22					29	22
P23	XT-CF20	Carbonadditive	3 × 3	0.44/0.5	40	38
P24				0.44/1.0		
P25				0.44/1.5		
P26				0.44/2.0		

**Table 2:** Degree of polarisation (DOP) at 90° for the second series of printed polarisers.

No. of Polariser	DOP at 90° [%]
P9	85.7
P10	86.1
P11	86.2
P12	86.5
P13	86.1
P14	83.2

**Table 3:** Normalised degree of polarisation [%] of WGP and polarisers with selected fill factor (FF) values for chosen frequencies.

No. of Polariser	FF [%]	150 GHz	200 GHz	300 GHz	400 GHz	500 GHz
WGP	-	99	99	98	98	98
P15	70	100	100	99	100	100
P16	60	100	100	99	100	99
P17	53	100	100	100	93	12
P18	46	100	100	74	58	−13
P19	38	99	89	11	18	−49
P20	30	97	13	2	25	−24
P21	25	32	−27	3	25	−6
P22	22	12	−28	−2	21	−8

**Table 4:** Transmittance $t_{⊥}$ [%] of WGP and polarisers with selected fill factor (FF) values for chosen frequencies.

No. of Polariser	FF [%]	150 GHz	200 GHz	300 GHz	400 GHz	500 GHz
WGP	-	89	93	86	84	80
P15	70	70	65	24	12	3
P16	60	75	64	18	12	3
P17	53	81	72	13	18	6
P18	46	87	75	18	29	10
P19	38	92	48	22	31	7
P20	30	85	41	30	45	19
P21	25	91	23	36	52	28
P22	22	71	24	34	36	37

## Data Availability

The raw data supporting the conclusions of this article will be made available by the authors on request.

## Abbreviations

The following abbreviations are used in this manuscript:

THz	terahertz
FDM	fused deposition modelling
FF	fill factor
LCD	liquid crystal display
WGP	wire-grid polariser
EM	electromagnetic
$t_{\|}$	parallel polarisation transmittance
$t_{⊥}$	perpendicular polarisation transmittance
DOP	degree of polarisation
THz-TDS	terahertz time-domain spectroscopy
COC	cyclic olefin copolymer
ASA	acrylonitrile styrene acrylate
PLA	polylactic acid
PETG	polyethylene terephthalate glycol
IMPATT	impact ionisation avalanche transit-time diode
*d*	thickness
